# In vivo evaluation of two tissue transglutaminase PET tracers in an orthotopic tumour xenograft model

**DOI:** 10.1186/s13550-018-0388-2

**Published:** 2018-05-25

**Authors:** Berend van der Wildt, Micha M. M. Wilhelmus, Wissam Beaino, Esther J. M. Kooijman, Robert C. Schuit, John G. J. M. Bol, John J. P. Breve, Ralf Pasternack, Adriaan A. Lammertsma, Albert D. Windhorst, Benjamin Drukarch

**Affiliations:** 1 Department of Radiology & Nuclear Medicine, VU University Medical Center, De Boelelaan 1085, 1081HV Amsterdam, The Netherlands; 2 Department of Anatomy & Neurosciences, VU University Medical Center, Amsterdam, The Netherlands; 3 Zedira GmbH, Darmstadt, Germany

**Keywords:** Transglutaminase type 2, Tissue transglutaminase, Positron emission tomography, MDA-MB-231

## Abstract

**Background:**

The protein cross-linking enzyme tissue transglutaminase (TG2; EC 2.3.2.13) is associated with the pathogenesis of various diseases, including cancer. Recently, the synthesis and initial evaluation of two high-potential radiolabelled irreversible TG2 inhibitors were reported by us. In the present study, these two compounds were evaluated further in a breast cancer (MDA-MB-231) tumour xenograft model for imaging active tissue transglutaminase in vivo.

**Results:**

The metabolic stability of [^11^C]**1** and [^18^F]**2** in SCID mice was comparable to the previously reported stability in Wistar rats. Quantitative real-time polymerase chain reaction analysis on MDA-MB-231 cells and isolated tumours showed a high level of TG2 expression with very low expression of other transglutaminases. PET imaging showed low tumour uptake of [^11^C]**1** (approx. 0.5 percentage of the injected dose per gram (%ID/g) at 40–60 min p.i.) and with relatively fast washout. Tumour uptake for [^18^F]**2** was steadily increasing over time (approx. 1.7 %ID/g at 40–60 min p.i.). Pretreatment of the animals with the TG2 inhibitor *ERW1041E* resulted in lower tumour activity concentrations, and this inhibitory effect was enhanced using unlabelled **2**.

**Conclusions:**

Whereas the TG2 targeting potential of [^11^C]**1** in this model seems inadequate, targeting of TG2 using [^18^F]**2** was achieved. As such, [^18^F]**2** could be used in future studies to clarify the role of active tissue transglutaminase in disease.

## Background

Transglutaminases comprise a family of enzymes responsible for the calcium-dependent intra- and intermolecular cross-linking of proteins between the side chains of glutamine and lysine residues, forming an epsilon-(gamma-glutaminyl)-lysine bond [1]. Tissue transglutaminase (TG2) is ubiquitously expressed and, under physiological conditions, plays a role in, e.g. apoptosis, cell differentiation and cell migration [2, 3]. The cross-linking activity of this enzyme is tightly regulated by various mechanisms. First, TG2 exists in two distinct conformations, referred to as closed and open conformations, respectively [4, 5]. Only in the open conformation, which is associated with high-calcium and low-guanosine diphosphate/guanosine triphosphate (GDP/GTP) concentrations, the active site cysteine residue is exposed and transamidation can be expected [5]. In the closed conformation, two consecutive C-terminal β-barrels sterically limit transamidation activity [4]. Second, the redox state of TG2 determines its catalytic activity, since a Cys370-Cys371 disulphide bridge, despite locking TG2 in its open conformation, hampers transamidation [6]. Finally, cross-linking activity of TG2 is regulated by formation of ternary protein complexes on the cell surface with extracellular matrix proteins, such as fibronectin and membrane-bound integrins [7]. Clearly, this multitude of regulatory mechanisms poses a challenge for assessing TG2 cross-linking activity in vivo. Often changes in TG2 expression levels or immunohistochemical detection of epsilon-(gamma-glutaminyl)-lysine bonds are used as ex vivo biomarkers of TG2 activity. Alternatively, transglutaminase mediated incorporation of systemically administered biotin-labelled amine substrates can be detected immunohistochemically after sacrificing the test animal [8, 9].

TG2 is strongly associated with the pathogenesis of cancer, celiac disease, and fibrotic and neurodegenerative diseases [10–15], in which its role is assumed to be related to its cross-linking activity. The fact that TG2 knock-out mice are phenotypically healthy in a stress-free environment has further boosted TG2 as a potential target for therapeutic intervention [16]. Nowadays, a wide array of TG2 inhibitors has been developed [17]. Nevertheless, further development of potent inhibitors towards clinical studies, for example by evaluation in animal models, has been limited. The availability of a validated TG2 PET tracer is likely to stimulate in vivo research of potent TG2 inhibitors, because it will allow monitoring of target engagement in vivo by novel TG2 inhibitors [18]. As a result, a deeper understanding of TG2 biology in various diseases might be obtained.

**Fig. 1:**
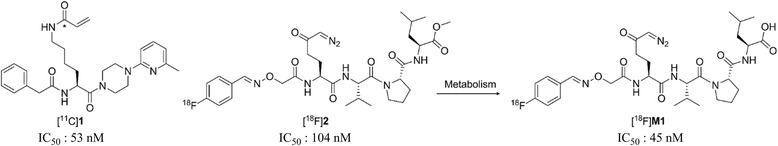
Chemical structures and IC_50_ values of the TG2 inhibitors [^11^C]**1**, [^18^F]**2**, together with that of its in vivo formed metabolite [^18^F]**M1** [19, 20]. The position of the carbon-11 label in [^11^C]**1** is depicted by an asterisk

Recently, carbon-11 and fluorine-18 labelled small molecule TG2 PET tracers have been developed by our group [19, 20]. [^11^C]**1** (Fig. 1, IC_50_ 53 nM) was selected out of three carbon-11 labelled TG2 inhibitors based on its superior metabolic stability [19]. In addition, [^18^F]**2**, a peptidic TG2 inhibitor (Fig. 1, IC_50_ 104 nM), was developed [20]. Despite being completely metabolised in vivo after just 15 min post injection, imaging with [^18^F]**2** was suggested as also the formed radiometabolite was previously shown to be an equipotent inhibitor of TG2 (IC_50_ 45 nM) [20]. Both selected compounds, [^11^C]**1** and [^18^F]**2**, were able to discriminate between active and inactive tissue transglutaminase in vitro and demonstrated specific and selective binding to MDA-MB-231 tumour sections, as assessed by in vitro autoradiography experiments. However, in vitro autoradiography assays do not necessarily reflect in vivo biology. Therefore, the aim of the present study was to determine whether these new tracers are able to target TG2 also in vivo. To this end, compounds [^18^F]**1** and [^18^F]**2** were evaluated in a mouse MDA-MB-231 tumour xenograft model.

## Methods

### Cell culture

MDA-MB-231 human breast cancer cells were purchased from American Type Culture Collection (Rockville, MD, USA). Cells were cultured at 37 °C, 5% CO_2_ in Dulbecco’s modified Eagle medium with 4.5 g/L glucose (Lonza, Basel, Switzerland) with HEPES supplemented with l-glutamine and fetal calf serum (5%).

### Xenograft model

Severe combined immunodeficient (SCID) female mice (6–8 weeks, 20 to 25 g, Charles River, Wilmington, MA, USA) were housed in sterile cages under standard conditions (24 °C, 60% relative humidity, 12-h light/dark cycles) and provided with water and food ad libitum. MDA-MB-231 cells (1 × 10^6^) were injected orthotopically in the fat pad of the second thoracic mammary glands (bilateral) [21]. Tumour dimensions were measured using a Vernier calliper, and tumour volume was calculated using the formula (*x*^2^ *y*)/2 (*x* and *y* being the width and length, respectively) for an ellipsoid. At 8 weeks after MDA-MB-231 cell injection, tumours reached the target size of 200 mm^3^. This study was performed according to national regulations and was approved by the Animal Experimentation Ethics Committee of the VU University Medical Center.

### QPCR analysis

Total messenger ribonucleic acid (mRNA) was isolated from MDA-MB-231 tumour cells or tumour tissue using Trizol Reagent (Invitrogen, Carlsbad, CA, USA) according to the manufacturer’s instructions. RNA was reverse-transcribed into complementary deoxyribonucleic acid (cDNA) using the High-Capacity cDNA Reverse Transcription kit (Applied Biosystems, Foster City, Ca, USA) using 0.5 μg oligo-dT primers according to the manufacturer’s instructions. For the subsequent quantitative real-time polymerase chain reaction (qPCR), the Power SYBR Green Master Mix (Applied Biosystems) was used. Primers were purchased from Eurogentec (Maastricht, Netherlands), and qPCR was performed in MicroAmp Optical 96-well Reaction Plates (Applied Biosystems) on a StepOnePlus Real-Time PCR system (Applied Biosystems). The reaction mixture (20 μL) was composed of 1 × Power SYBR Green buffer (Applied Biosystems), 3.75 pmol of each primer (see Table 1 for primer details), and 12.5 ng cDNA. The thermal cycling conditions were an initial 10 min at 95 °C followed by 50 cycles of 15 s at 95 °C and 1 min at 60 °C. The specificity of the reaction was checked by means of melt curve analysis. Relative expression levels of the target genes were determined by LinRegPCR software (version 2014.3; website: http://www.hfrc.nl) using the following equation *N*_0_ = *N*_q_/(*E*^*C*_q_) (*N*_0_ = target quantity, *N*_q_ = fluorescence threshold value, *E* = mean PCR efficiency per amplicon, *C*_q_ = threshold cycle) [22], after which the value was normalised to the expression level of the reference gene glyceraldehyde-3-phosphate-dehydrogenase (GAPDH) using the following formula (*N*_o, gene of interest_/N_o, GAPDH_). Results are expressed as gene expression relative to GAPDH ± standard deviation (*n* = 4 for both tumour cells and tumour tissue measurements).

**Table 1:** Primer sequences used for qPCR analysis

Gene	NCBI reference sequence	Forward (5′→3′)	Reverse (3′→5′)	Amplicon size (base pairs)
GAPDH	NM_002046.3	TCAAGGGCATCCTGGGCTAC	CGTCAAAGGTGGAGGAGTGG	81
TGM1	NM_000359.2	CAATGTCTCAGGCCACGTC	CCAGTAACGTGAGGGAGAGG	95
TGM2	NM_004613.2	AGAGGAGCGGCAGGAGTATG	AGGATCCCATCTTCAAACTGC	111
TGM3	NM_003245.3	AACCTGAAGATCGACGTGC	CAGTTGCTTGGTGCCACTC	93
TGM5	NM_201631.3	TCCTGGTGAACAAGATCATC	GTATGGAGAGTGGCTGGTTC	90
F13A1	NM_000129.3	TCCGCAGAGGGCAGTCTTTC	CCTGTGGGTAGCGACCAATGA	105

### Radiotracer synthesis

#### (*S*)-*N*-(6-(4-(6-methylpyridin-2-yl)piperazin-1-yl)-6-oxo-5-(2-phenylacetamido)hexyl)[^11^C]acrylamide ([^11^C]1)

**Scheme 1:**
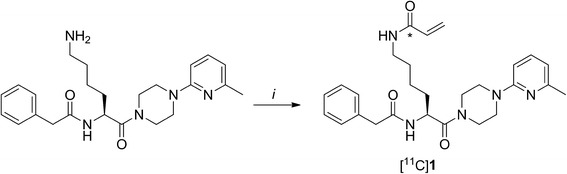
Radiosynthesis of [^11^C]**1**. Reagents and conditions: (i) [^11^C]CO, vinyl iodide, Pd_2_(dba)_3_, PPh_3_, tetrahydrofuran (THF), 5 min, 100 °C

[^11^C]**1** was synthesised as previously described (Scheme 1) [19] and was obtained in 20–28% decay corrected (d.c) yield based on [^11^C]CO with a molar activity of 144 ± 22 GBq · μmol^−1^ and a radiochemical purity > 99% (*n* = 4). The identity of the product was confirmed by analytical high-performance liquid chromatography (HPLC) analysis by co-injection of radiolabelled and unlabelled **1** using a Phenomenex Luna Phenyl-hexyl column (5 μm, 250 mm × 4.6 mm) with H_2_O/acetonitrile (MeCN), 7:3 (*v*/*v*) as eluent, with a flow rate of 1 mL/min, retention time (*R*_t_) 9.0 min.

#### (*S*)-methyl 2-((*S*)-1-((7S,10S,E)-7-(4-diazo-3-oxobutyl)-1-(4-[^18^F]fluorophenyl)-10-isopropyl-5,8-dioxo-3-oxa-2,6,9-triazaundec-1-en-11-oyl)pyrrolidine-2-carboxamido)-4-methylpentanoate ([^18^F]2)

**Scheme 2:**
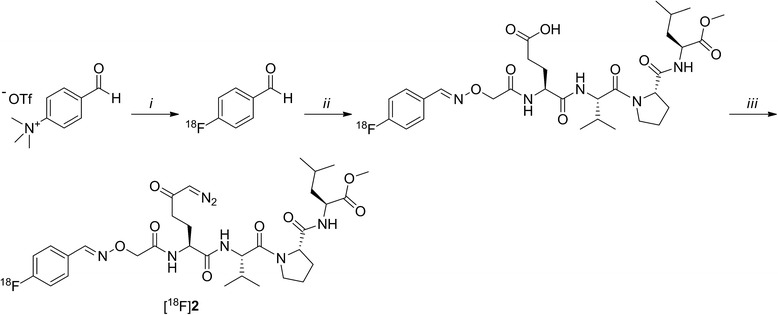
Radiosynthesis of [^18^F]**2**. Reagents and conditions: (i) K_2_CO_3_, [^18^F/K_222_], DMF, 15 min, 100 °C; (ii) (*S*)-4-(2-(aminooxy)acetamido)-5-(((*S*)-1-((*S*)-2-(((*S*)-1-methoxy-4-methyl-1-oxopentan-2-yl)carbamoyl)pyrrolidin-1-yl)-3-methyl-1-oxobutan-2-yl)amino)-5-oxopentanoic acid, trifluoroacetic acid (TFA), *N*,*N*-dimethylformamide (DMF)/THF, 30 min, 50 °C; (iii) isobutylchloride, *N*-methylmorpholine, DMF/THF, 5 min, − 10 °C, then CH_2_N_2_ (0.5 M in Et_2_O), 30 min, − 10 °C to rt

[^18^F]**2** was synthesised as previously described (Scheme 2) [20] and was obtained in 3–4% d.c. yield, calculated at the time of the start of the synthesis, with a molar activity of 111 ± 28 GBq · μmol^−1^ and a radiochemical purity > 98% (*n* = 4). The identity of the product was confirmed by analytical HPLC analysis by co-injection of radiolabelled and unlabelled **2** using a Phenomenex Luna Phenyl-hexyl column (5 μm, 250 mm × 4.6 mm) with H_2_O/MeCN/TFA, 60:40:0.1 (*v*/*v*/*v*) as eluent, with a flow rate of 1 mL/min, *R*_t_ 28 min.

### Metabolite analysis

Healthy female SCID mice (20–25 g, Harlan, Horst, The Netherlands) were injected in the tail vein with [^11^C]**1** or [^18^F]**2** (20 MBq) under isoflurane anaesthesia (2% in O_2_ at 1 L · min^−1^). Mice were sacrificed at 15 and 45 min post injection (*n* = 3 for each time-point) by cervical dislocation. Blood (~ 0.5–1 mL) was withdrawn by heart puncture, collected in a heparin-coated tube and centrifuged at 5000 RPM to separate plasma from blood cells. Solid phase extraction (SPE) cartridges (tC18, Waters, Milford, MA, USA) were preconditioned by washing with methanol (MeOH, 6 mL) and H_2_O (two times 6 mL). Plasma (~ 0.2 mL) was mixed with 0.15 M HCl (0.2 mL) and loaded on the tC18 cartridge. The polar fraction was obtained by elution of the cartridge with H_2_O (3 mL), the non-polar fraction by subsequent elution with MeOH (1.5 mL) and H_2_O (0.5 mL). Both fractions were counted for radioactivity in a gamma counter. The percentage of intact tracer in the non-polar fraction was determined by online and offline HPLC analysis (Gemini C18 column (5 μm, 10 mm × 250 mm, Phenomenex, Torrance, CA, USA) with eluent MeCN (A) and 0.1% TFA acid in H_2_O (B) as eluent according to the following schemes; For [^11^C]**1**: 0 min, 70% B at 0.25 mL · min^−1^; 0.5 min, 70% B at 3.5 mL · min^−1^; 9.0 min, 10% B at 3.5 mL · min^−1^; 12.0 min, 10% B at 3.5 mL · min^−1^; 12.5 min, 70% B at 3.5 mL · min^−1^; 14.5 min, 70% B at 3.5 mL · min^−1^ and 15 min, 70% B at 0.25 mL · min^−1^ or for [^18^F]**2**: 0 min, 80% B at 0.25 mL · min^−1^; 0.5 min, 80% B at 3 mL · min^−1^; 6.0 min, 30% B at 3 mL · min^−1^; 12.0 min, 30% B at 3 mL · min^−1^; 12.5 min, 80% B at 3.0 mL · min^−1^; 14.5 min, 80% B at 3.0 mL · min^−1^; 15.0 min, 80% B at 0.25 mL · min^−1^ (*R*_t_ = 11.5 min). Offline HPLC analysis was performed when the online HPLC analysis yielded a low signal to noise ratio. For offline analysis, HPLC fractions (30 s per fraction) were collected and counted for radioactivity (WIZARD 2480 Compugamma, PerkinElmer, Waltham, MA, USA). The counting results were plotted to generate the corresponding HPLC chromatograms. Data were expressed as percentage of intact tracer, polar metabolites, and non-polar metabolites ± standard deviation.

### Metabolite characterisation

To healthy SCID mice (*n* = 2) 10 MBq [^18^F]**2** diluted with 50 μg (2.5 mg · kg^−1^) of unlabelled **2** was administered, corresponding to a molar activity of 0.14 GBq · μmol^−1^. At 30 min post injection, animals were sacrificed under isoflurane anaesthesia (2% in O_2_ at 1 L · min^−1^) by cervical dislocation. Blood (~ 1 mL) was collected by heart puncture. Blood plasma was separated using a SPE procedure as described in the “Metabolite analysis” section (vide supra). The non-polar fraction was analysed by HPLC and Liquid chromatography–tandem mass spectrometry (LC-MS/MS) in multiple reaction monitoring (MRM) mode. LC-MS/MS analysis was performed on a Jasco system (Easton, PA, USA) with an AB Sciex QTRAP 5500 mass spectrometer (Concorde, Ontario, Canada). The Jasco system consisted of two pumps (X-LC 3180PU), a degasser (X-LC 3080DG), a mixer (X-LC 3080MX), a column oven (X-LC 3080CO), and databoxes LV 2080-03 and LC-Net II/ACD. A Kinetex C18 column (1.7 u, 100 A, 100 × 2.10 mm, Phenomenex, Torrance, CA, USA) at 25 °C was used for chromatographic separation with eluent MeCN (A) and 0.1% formic acid in H_2_O (B) according to the following scheme: 0 min, 90% B at 0.5 mL · min^−1^; 3 min, 10% B at 0.5 mL · min^−1^; 5 min, 10% B at 0.5 mL · min^−1^; 5.5 min 90% B at 0.5 mL · min^−1^. Capillary potential was set at 5.5 kV, source temperature at 100 °C, and desolvation temperature at 750 °C. MRM Q1 and Q3 set at 696.2–418.0 m/z for detection of compound 2 and 682.1–626.3 m/z for detection of **M1**.

### Chemical synthesis

#### General

All reagents were obtained from commercial sources (Sigma Aldrich, St. Louis, USA). Solvents were obtained from Biosolve (Valkenswaard, the Netherlands) and used as received unless stated otherwise. Dichloromethane (DCM) and DMF were dried over activated 3 Å molecular sieves. THF was first distilled from LiAlH_4_ and then stored on activated 3 Å molecular sieves. Reaction monitoring by thin-layer chromatography was performed on pre-coated silica 60 F254 aluminium plates (Merck, Darmstadt, Germany). Spots were visualised by UV light or ninhydrin. Evaporation of solvents was performed under reduced pressure at 40 °C using a rotary evaporator. Flash column chromatography was performed manually on Silica gel 60 Å (Merck, Darmstadt, Germany). Nuclear magnetic resonance (NMR) spectroscopy was performed using a Bruker (Billerica, MA, USA) Avance 250 (250.13 MHz for ^1^H and 62.90 MHz for ^13^C) or an Avance 500 (500.23 MHz for ^1^H and 125.78 MHz for ^13^C) with chemical shifts (δ) reported in parts per million (ppm) relative to the solvent (chloroform (CDCl_3_), ^1^H 7.26 ppm, ^13^C 77.16 ppm). Electrospray ionisation-high resolution mass spectrometry (ESI-HRMS) was carried out using a Bruker microTOF-Q instrument in positive ion mode (capillary potential of 4500 V).

#### (*S*)-methyl 2-((*S*)-1-((7S,10S)-7-(4-diazo-3-oxobutyl)-1-(4-fluorophenyl)-10-isopropyl-5,8-dioxo-3-oxa-2,6,9-triazaundec-1-en-11-oyl)pyrrolidine-2-carboxamido)-4-methylpentanoate (2)

The synthesis of unlabelled **2** was performed as published elsewhere [20].

**Scheme 3:**
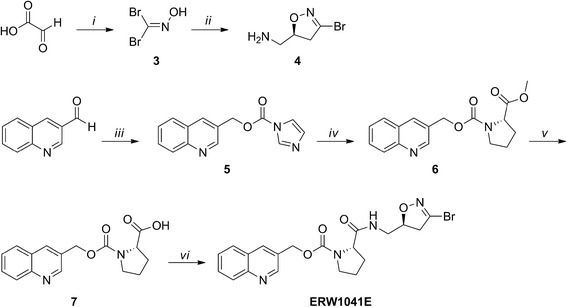
Synthesis of *ERW1041E*. Reagents and conditions: (i) hydroxylamine hydrochloride, H_2_O, 2 h, room temperature (rt), then NaHCO_3_, Br_2_, 16 h, 0 °C to rt, 52%; (ii) allylamine hydrochloride, KHCO_3_, H_2_O, 3 h, rt, 50%; (iii) LiBH_4_, ethanol/THF, 2 h, − 80 °C, then CDI, MeCN, 2 h, rt, 78%; (iv) proline methyl ester hydrochloride, D*i*PEA, DCM/DMF, 16 h, rt, 60%; (v) NaOH, dioxane/MeOH/H_2_O, 2 h, rt, 89%; (vi) amine **4**, EDC, HOBt, *N*-methylmorpholine, DMF, 2 h, rt, 96%

Synthesis of *ERW1041E* was performed according to published procedures (Scheme 3) [9]. Analytical characterizations were in accordance with reported values [9, 23].

#### (*S*)-(3-Bromo-4,5-dihydroisoxazol-5-yl)methanamine (4)

A solution of 50% glyoxylic acid (15.0 mL, 135 mmol) and hydroxylamine hydrochloride (9.40 g, 135 mmol) in H_2_O (50 mL) was stirred for 2 h at room temperature (rt). Sodium bicarbonate was added (22.7 g, 270 mmol), and the resulting solution was cooled on ice. Bromine was added dropwise (9.00 mL, 176 mmol), and the resulting solution was stirred overnight at rt. The mixture was extracted with DCM (three times 100 mL). The combined organic fractions were dried on Na_2_SO_4_, filtered and carefully concentrated in vacuo to afford dibromoformaldoxime **3** as a white solid (14.1 g, 52%), which was immediately used as such in the subsequent reaction. To a solution of allylamine hydrochloride (7.0 g, 75 mmol) in H_2_O (100 mL) was added dibromoformaldoxime **3** (10 g, 49 mmol). To this solution was added dropwise a solution of KHCO_3_ (6.0 g, 60 mmol) in H_2_O (50 mL) over a 3-h period while stirring vigorously at rt. The resulting solution was stirred for another 3 h prior to dilution with saturated NaHCO_3_ (100 mL). After extraction with DCM (three times 100 mL), the combined organic fractions were dried on Na_2_SO_4_ and concentrated in vacuo. Flash column chromatography (DCM/MeOH, 9:1 (*v*/*v*)) afforded racemic (3-bromo-4,5-dihydroisoxazol-5-yl) methanamine as a yellow oil (5.1 g, 58%). Enantiomeric enrichment was performed as follows: to a solution of racemic (3-bromo-4,5-dihydroisoxazol-5-yl) methanamine (2.3 g, 12.9 mmol) and l-mandelic acid (1.0 g, 6.4 mmol) in hot MeOH was added dropwise hot isopropanol. The solution was allowed to cool to rt and left for 16 h. The solids were filtered off and recrystallization was performed out of a hot MeOH/isopropanol mixture until white crystalline needles were formed. After filtration, the needles were dissolved in saturated NaHCO_3_ and the solution was extracted with DCM (three times 20 mL). After drying on Na_2_SO_4_, filtration and concentration in vacuo, product **4** was obtained as a colourless oil 1.0 g, 87%). ^1^H NMR (500 MHz, CDCl_3_): δ 4.74 (m, 1H), 3.25 (dd, 1H, *J* = 17.4, 10.7 Hz), 3.08 (dd, 1H, *J* = 17.0, 7.8 Hz), 3.01 (dd, 1H, *J* = 13.5, 3.8 Hz) and 2.58 (dd, 1H, *J* = 13.5, 5.7 Hz); ^13^C NMR (125 MHz, CDCl_3_): δ 137.52, 83.11, 44.99 and 43.85; ESI-HRMS calculated for C_4_H_7_BrN_2_O: 177.9742, found: 178.9811 [M + H]^+^.

#### Quinolin-3-ylmethyl 1*H*-imidazole-1-carboxylate (5)

A solution of quinolone-3-carbaldehyde (2.50 g, 15.9 mmol) in ethanol/THF (20 mL) was cooled to − 80 °C prior to the addition of LiBH_4_ (0.34 g, 16 mmol). After stirring for 2 h, the mixture was quenched with 1 M HCl (100 mL) and allowed to reach rt. The resulting solution was diluted with saturated NaHCO_3_ (100 mL) and extracted ethyl acetate (EtOAc) (three times 50 mL). The collected EtOAc fractions were dried on Na_2_SO_4_, filtered and concentrated in vacuo to obtain quinolyn-3-ylmethanol as yellow oil. Quinolyn-3-ylmethanol (2.40 g, 15.1 mmol) was dissolved in MeCN (10 mL) and carbonyldiimidazole (4.90 g, 30.2 mmol) was added to this solution. After stirring for 2 h at rt, the white precipitate was filtered and carefully washed with ice-cold acetonitrile (10 mL) obtaining compound **5** as a white solid (2.98 g, 78%). ^1^H NMR (250 MHz, CDCl_3_): δ 9.01 (d, 1H, *J* = 2.2 Hz), 8.27 (d, 1H, *J* = 2.1 Hz), 8.17–8.13 (m, 2H), 7.88 (dd, 1H, *J* = 8.1, 1.2 Hz), 7.79 (m, 1H), 7.61 (m, 1H), 7.44 (m, 1H), 7.07 (dd, 1H, *J* = 1.8, 0.9 Hz) and 5.62 (s, 2H); ^13^C NMR (125 MHz, CDCl_3_): δ 150.72, 148.65, 148.41, 137.25, 136.89, 131.01, 130.63, 129.53, 128.14, 127.55, 127.53, 126.87, 117.24 and 67.61.

#### 2-Methyl 1-(quinolin-3-ylmethyl) (*S*)-pyrrolidine-1,2-dicarboxylate (6)

To a solution of l-proline methyl ester hydrochloride (1.3 g, 7.9 mmol) and D*i*PEA (1.4 mL, 7.9 mmol) in DCM/DMF (1:1 (*v*/*v*), 10 mL) was added compound **5** (2.0 g, 7.9 mmol) in DCM (5 mL) and the solution was stirred at rt. for 16 h. After concentrating in vacuo, the residue was dissolved in EtOAc and purified by flash column chromatography (hexane/EtOAc 3:2) obtaining compound **6** as a colourless oil (1.50 g, 60%). ^1^H NMR (250 MHz, CDCl_3_, mixture of rotamers): δ 8.93 and 8.87 (2× d, 1H, *J* = 2.2 Hz), 8.11 (dt, 1H, *J* = 12.1, 1.4 Hz), 7.84–7.79 (m, 1H), 7.75–7.66 (m, 1H), 7.58–7.51 (m, 1H), 5.41–5.20 (m, 2H), 4.37 (dt, 1H, *J* = 8.9, 3.8 Hz), 3.73–3.45 (m, 5H) and 2.27–1.85 (m, 5H); ^13^C NMR (500 MHz, CDCl_3_, mixture of rotamers): δ 173.21, 173.05, 154.66, 154.04, 150.82, 150.61, 147.92, 147.88, 135.53, 135.26, 129.77, 129.74, 129.73, 129.52, 129.48, 129.35, 128.00, 127.96, 127.73, 127.00, 64.89, 64.78, 59.31, 58.92, 52.40, 52.32, 47.10, 46.57, 31.01, 29.96, 24.40 and 23.57; ESI-HRMS: calculated for C_17_H_18_N_2_O_4_: 314.1267, found: 315.1348; 337.1158 [M + H]^+^, [M + Na]^+^.

#### ((Quinolin-3-ylmethoxy)carbonyl)-l-proline (7)

A solution of compound **6** (1.00 g, 3.18 mmol) in Tesser’s base (dioxane/MeOH/4 M NaOH, 6:4:2 (*v*/*v*/*v*) 10 mL) was stirred for 2 h at rt prior to acidification to pH 4 with 1 M HCl. The solution was extracted with DCM (six times 20 mL). The combined organic fractions were dried on Na_2_SO_4_, filtered and concentrated in vacuo, obtaining compound **7** as a white solid (0.85 g, 89%). ^1^H NMR (500 MHz, CDCl_3_, mixture of rotamers): δ 11.32 (br s, 1H), 8.93 (s, 1H), 8.27 (s, 1H), 7.83–7.67 (m, 2H), 7.56–7.41 (m, 2H), 5.56 (d, 1H, *J* = 13 Hz), 5.09 (d, 1H, *J* = 12.6 Hz), 4.47–4.38 (m, 1H), 3.68–3.61 (m, 1H), 3.56–3.47 (m, 1H), 2.32–2.10 (m, 2H) and 2.01–1.85 (m, 2H); ^13^C NMR (500 MHz, CDCl_3_, mixture of rotamers): δ 175.58, 175.25, 154.97, 154.21, 149.16, 148.74, 144.34, 138.05, 137.38, 130.75, 130.70, 130.04, 128.07, 127.98, 127.89, 127.70, 127.61, 127.32, 125.98, 64.53, 64.19, 59.78, 59.45, 47.18, 46.72, 31.03, 29.88, 24.40 and 23.71; ESI-HRMS: calculated for C_16_H_16_N_2_O_4_: 300.1110, found: 301.1189; 323.1006 [M + H]^+^, [M + Na]^+^.

#### Quinolin-3-ylmethyl (S*)-*2-((((*S*)-3-bromo-4,5-dihydroisoxazol-5-yl)methyl)carbamoyl)pyrrolidine-1-carboxylate (ERW1041E)

A solution of carboxylic acid **7** (0.40 g, 1.3 mmol), amine **4** (0.24 g, 1.3 mmol), 1-ethyl-3-(3-dimethylaminopropyl)carbodiimide (EDC, 0.28 g, 1.5 mmol), hydroxybenzotriazole (HOBt, 0.20 mg, 1.3 mmol) and *N*-methylmorpholine (0.15 mL, 1.3 mmol) in DMF (5 mL) was stirred for 2 h. The solution was concentrated in vacuo, and the residue was purified by flash column chromatography (DCM/MeOH, 25:1 (*v*/*v*)), obtaining the product as colourless solid (0.59 g, 96%). ^1^H NMR (500 MHz, CDCl_3_, mixture of rotamers): δ 8.85 (m, 1H), 8.09 (m, 2H), 7.78 (m, 1H), 7.67 (m, 1H), 7.52 (m, 1H), 7.07–6.91 (m, 1H), 5.35–5.21 (m, 2H), 4.75–4.54 (m, 1H), 4.29 (m, 1H), 3.65–3.35 (m, 4H), 3.18–2.80 (m, 2H) and 2.12–1.83 (m, 4H); ^13^C NMR (125 MHz, CDCl_3_, mixture of rotamers): δ 173.19, 172.72, 155.32, 154.49, 150.63, 147.72, 138.00, 137.44, 135.56, 129.77, 129.22, 129.10, 127.91, 127.55, 126.94, 80.49, 80.21, 65.07, 64.89, 60.86, 60.63, 47.55, 47.05, 43.80, 43.62, 41.78, 41.23, 31.47, 29.59, 24.51 and 23.59; ESI-HRMS: calculated for C_20_H_21_BrN_4_O_4_: 460.0746, found: 461.0805, 483.0620 [M + H]^+^, [M + Na]^+^.

### PET imaging

Dynamic PET imaging was performed using dedicated small animal NanoPET/CT and NanoPET/MR scanners (Mediso Ltd., Hungary, Budapest) [24, 25] with identical PET components. Mice (*n* = 4 per group) were anaesthetized with 4 and 2% isoflurane in 1 L · min^−1^ oxygen for induction and maintenance, respectively. Mice were positioned on the scanner bed, and the respiratory rate was monitored for the duration of the scan, adjusting anaesthesia when required. A dynamic PET scan was acquired immediately after intravenous (i.v.) administration (tail vein) of 5 MBq [^11^C]**1** or [^18^F]**2**. For blocking experiments, mice were injected subcutaneously with *ERW1041E* (50 mg · kg^−1^) dissolved in 20% dimethylsulfoxide in 0.9% saline, 30 min prior to the tracer injection. An additional blocking experiment was performed by co-administration of compound **2** (50 μg, 75 nmol) and [^18^F]**2**, which corresponded with a molar activity of 0.07 GBq · μmol^−1^. PET scans were acquired in list mode and rebinned into the following frame sequence: 4 × 5, 4 × 10, 2 × 30, 3 × 60, 2 × 300, 1 × 600, 1 × 900 and 1 × 1200 s. In addition, a static [^18^F]2-fluoro-2-deoxy-d-glucose ([^18^F]FDG) scan was acquired for 30 min immediately after [^18^F]FDG administration (10 MBq, tail vein). At least a 24-h time interval between [^18^F]FDG scans and [^11^C]**1** or [^18^F]**2** scans was maintained. Reconstruction was performed with a fully 3-dimensional (3D) reconstruction algorithm using four iterations and six subsets, resulting in an isotropic 0.4-mm voxel dimension. Images were analysed using the freely available AMIDE-software version 1.0.4 (retrieved from https://sourceforge.net/projects/amide/files/amide/1.0.4). Regions of interest (ROIs) were drawn around the tumour tissue and leg muscle. Results are expressed as percentage injected dose per gram (%ID/g). Error bars indicate standard deviation. After PET scanning experiments, animals were sacrificed by cervical dislocation, tumours were isolated, and stored at − 80 °C until further use.

### Haematoxylin and eosin staining

MDA-MB-231 tumour sections (10 μm) were dried and fixed with acetone (100%) for 10 min and subsequently dried at rt. Sections were then rehydrated in Tris buffered saline (TBS; two times 5 min) and demiwater (5 min) and stained with Mayer’s haematoxylin solution (3 min) followed by rinsing with tap water (5 min). The sections were stained with 1% eosin Y solution (10–30 s) followed by dehydrating by sequential dipping in ethanol (70, 90, 96, 100 and 100%) and xylene. Sections were then mounted with coverslips using Entellan. Microscopy images were obtained using a Leica DN5000B microscope (Leica Microsystems, IL, USA).

### Immunohistochemical staining

Immunohistochemical staining of TG2 was performed as described previously with minor modifications [19]. Fresh frozen MDA-MB-231 tumour sections (10 μm) were dried and fixed with acetone (100%) for 10 min, dried at rt and subsequently rehydrated using TBS (three times 5 min). Endogenous peroxidase activity was blocked with 0.3% H_2_O_2_ and 0.1% NaN_3_ in TBS for 15 min and then washed with TBS (three times 5 min). After blocking with 3% bovine serum albumin (BSA) in TBS with 0.5% TritonX-100 (TBS-T) for 20 min, incubation with polyclonal goat anti-guinea pig TG2 antibody (Upstate, Merck Millipore, Billerica, MA, USA) in TBS-T with 3% BSA was performed overnight at 4 °C (dilution 1:4000). A negative control experiment was performed by omitting the primary antibody (results not shown). Sections were then washed with TBS (three times 5 min) prior to incubation with biotinylated donkey anti-goat secondary antibody (Jackson ImmunoResearch Laboratories Inc., West Grove, PA, USA, dilution 1:400) for 2 h at rt. Excess antibody was removed by washing with TBS (three times 5 min), and sections were incubated for 1 h with horseradish peroxidase avidin-biotin complex. After sequential washing with TBS (two times 5 min) and Tris-HCl (5 min), peroxidase-mediated [1,1′-biphenyl]-3,3′,4,4′-tetraamine (DAB) oxidation was used for visualising TG2. Colouring was monitored ad oculos. After colouring was satisfactory, sections were washed sequentially with Tris-HCl (two times 5 min) and water (5 min). Dehydration was carried out by sequential dipping in ethanol (70, 90, 96, 100 and 100%) and xylene, and sections were mounted with coverslips using Entellan. Microscopy images were obtained using a Leica DN5000B microscope (Leica Microsystems, IL, USA).

### Histochemical staining

Histochemical staining of active TG2 was performed as described previously [19]. MDA-MB-231 tumour sections (10 μm) were pre-incubated in 100 mM Tris-HCl buffer (pH 7.4), 5 mM CaCl_2_, 1 mM dithiothreitol (DTT) rt (20 min). As negative control, the selective TG2 inhibitor **Z006** was added to this solution at a final concentration of 100 μM (results not shown). Next, sections were incubated in 100 mM Tris-HCl buffer (pH 7.4), 5 mM CaCl_2_, 1 mM DTT and the TG2 amine donor substrate 5-(biotinamido)pentylamine (BAP; 0.05 mM) at 37 °C for 30 min. After a short wash with TBS and water, the sections were dried at rt. Then, the sections were fixed with acetone for 10 min followed by washing with Tris buffered saline (TBS). Sections were blocked with 0.1% NaN_3_ and 0.3% H_2_O_2_ in TBS for 15 min, washed with TBS (three times 5 min) and incubated for 1 h with horseradish peroxidase avidin-biotin complex. After washing (two times 5 min TBS, then 5 min Tris-HCl), peroxidase was developed by addition of DAB and colouring was monitored ad oculos. Nuclear staining using haematoxylin was performed. Sections were washed with Tris-HCl and water, dehydrated by sequential dipping in ethanol (70, 90, 96, 100 and 100%) and xylene, and mounted with coverslips using Entellan. Microscopy images were obtained using a Leica DN5000B microscope (Leica Microsystems, IL, USA).

### Autoradiography

Autoradiography was performed essentially as described previously [20]. MDA-MB-231 tumour sections (10 μm) were washed three times with 50 mM Tris-HCl buffer (pH 7.4) for 5 min. Sections were dried under a gentle air flow before incubation for 30 min with [^18^F]**2** (0.1 MBq · mL^−1^) in 5 mM Tris-HCl, pH 7.4, 5 mM CaCl_2_, 1 mM DTT. As a negative control inhibitor **1** was added to this incubation solution at 100 μM (results not shown). Washing was performed using 5 mM Tris-HCl (three times) followed by dipping in ice cold water. After drying in an air stream, tumour sections were exposed to a phosphorimaging screen (GE Healthcare, Buckinghamshire, UK) for 15 min and developed on a Typhoon FLA 7000 phosphor imager (GE Healthcare, Buckinghamshire, UK). Visualisation of binding was performed using ImageQuantTL v8.1.0.0 (GE Healthcare, Buckinghamshire, UK).

### Statistical analysis

Where relevant, statistical analysis was performed using either a one-tailed paired Student’s *t* test or a two-tailed paired Student’s *t* test with a confidence interval of 95%.

## Results

### mRNA expression of various human transglutaminases in MDA-MB-231 cells and tumour xenograft tissue

**Fig. 2:**
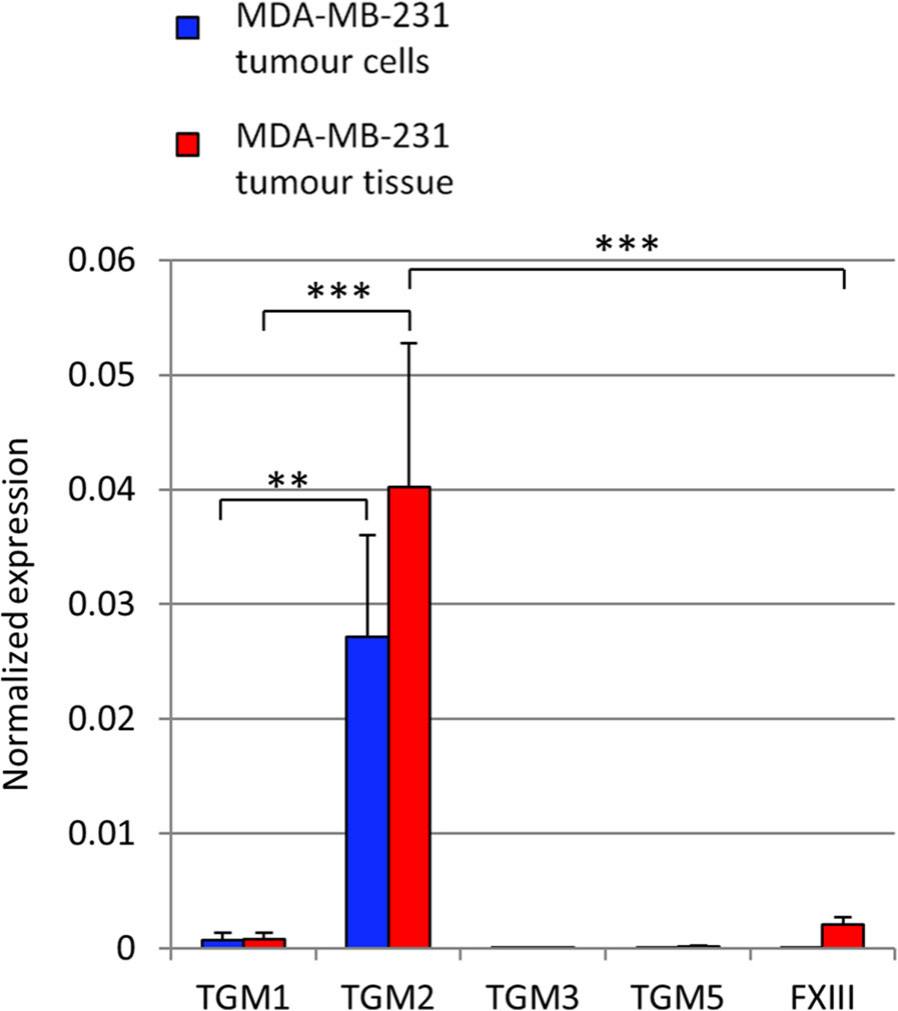
Transglutaminase mRNA expression in MDA-MB-231 tumour cells and in MDA-MB-231 tumour tissue. Results are expressed as relative values to GAPDH mRNA expression ± standard deviation (*n* = 4; **p < 0.005; ***p < 0.0005)

The expression of various tumour-related transglutaminases was determined by means of qPCR on both in vitro cultured MDA-MB-231 tumour cells and ex vivo MDA-MB-231 tumour material obtained after tumour inoculation in SCID mice. RNA expression levels of transglutaminase types 1–3 and 5 and blood coagulation factor XIII (TG1, TG2, TG3, TG5 and FXIII) were quantitatively determined relative to GAPDH RNA expression (Fig. 2). In MDA-MB-231 tumour cells as well as in tumour tissue, TG2 mRNA was most abundant. Low levels of TG1 mRNA were observed in both cells and tissue, whereas TG3 and TG5 mRNA were absent. FXIII mRNA was absent in cultured MDA-MB-231 tumour cells, whereas in tumour tissue low levels, relative to TG2 mRNA, were found.

### Metabolite analysis of [^11^C]1 and [^18^F]2 in SCID mice

**Fig. 3:**
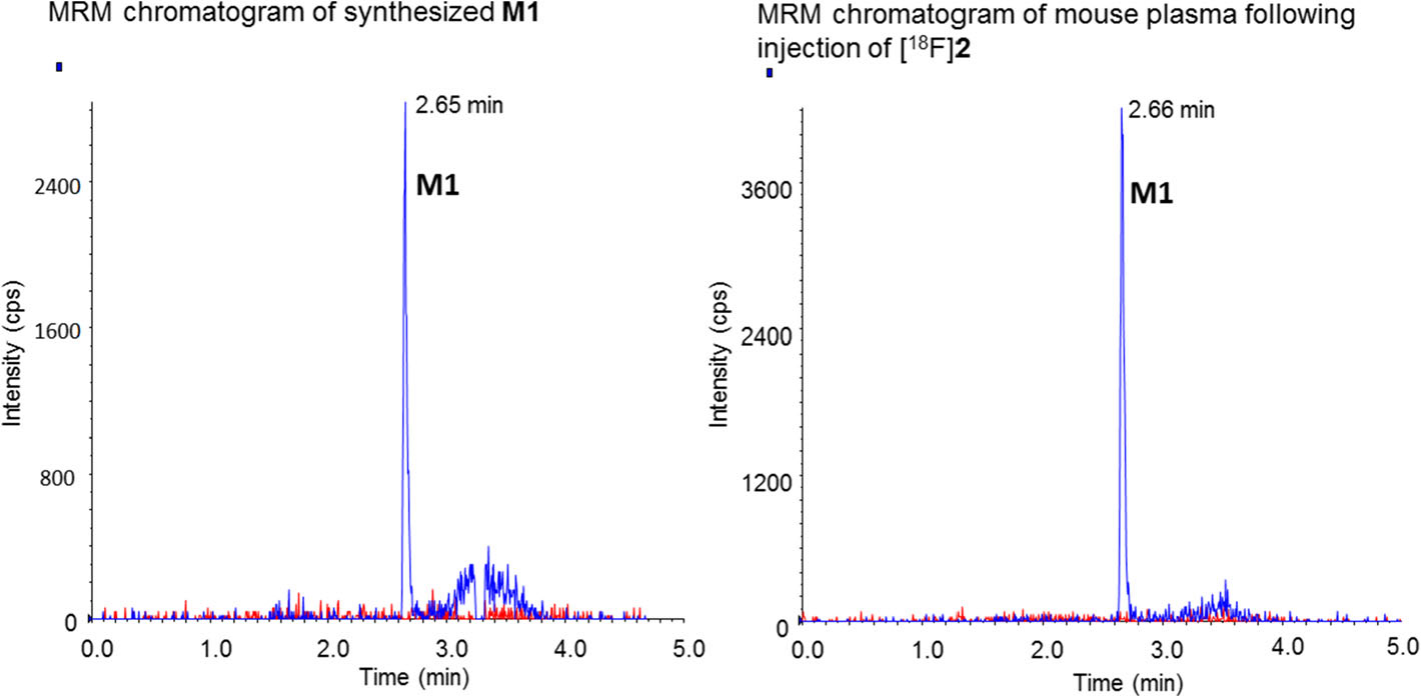
LC-MS/MS spectra following carrier added administration of [^18^F]**2** to SCID mice. MRM mass transitions 696.2–418.0 m/z (corresponding to **2**, red line) and 682.2–626.3 m/z (corresponding to **M1**, blue line). Left chromatogram: LC-MS/MS analysis of synthesised **M1**. Right chromatogram: LC-MS/MS analysis of mouse plasma after administration of [^18^F]**2**. LC-MS/MS analysis indicated full conversion of **2** to **M1**

In SCID mice, compound [^11^C]**1** demonstrated moderate metabolism, with 24% intact tracer after 45 min (Table 2). Mainly polar metabolites were formed. [^18^F]**2** was fully metabolised to a single metabolite after 45 min. The metabolite was identified by LC-MS/MS analysis as the demethylated parent **M1** (Fig. 3).

**Table 2:** Plasma metabolite profile of [^11^C]**1** and [^18^F]**2** following i.v. administration in healthy SCID mice and previously obtained results following i.v. administration in healthy Wistar rats [19, 20]

	[^11^C]**1**		[^18^F]**2**	
	Mouse	Rat	Mouse	Rat
15 min				
Intact (%)	56 ± 14	65 ± 7	20 ± 1	1 ± 1
Non-polar (%)	24 ± 15	13 ± 4	76 ± 3	98 ± 1
Polar (%)	20 ± 5	21 ± 3	1 ± 0.2	2 ± 0
45 min				
Intact (%)	24 ± 1	29 ± 11	3 ± 2	0 ± 0*
Non-polar (%)	28 ± 8	6 ± 3	95 ± 2	92 ± 3*
Polar (%)	47 ± 7	65 ± 12	2 ± 1	8 ± 3*

Following i.v. administration in healthy animals, anaesthetised using isoflurane, blood plasma was obtained at 15 and 45 min. Blood plasma was separated into a polar and non-polar fraction using a SPE method. Non polar fractions were analysed on HPLC. Results are expressed as average percentage of total blood plasma activity ± standard deviation (*n* = at least three per data point). *Animals were sacrificed at 60 min post injection.

### PET scanning

**Fig. 4:**
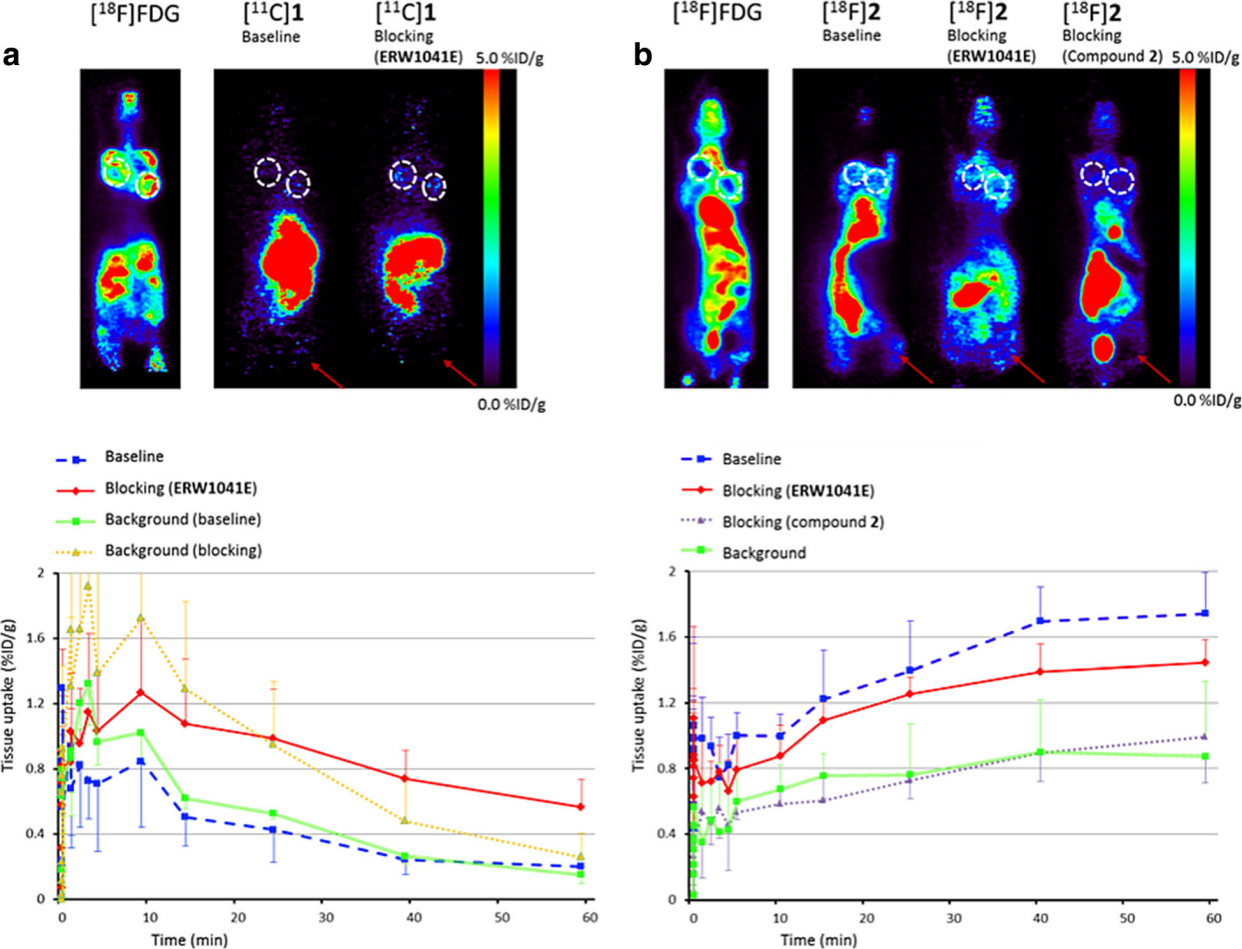
PET images and TACs of [^11^C]**1** or [^18^F]**2** in MDA-MB-231 tumour bearing SCID mice (*n* = 4 per group). Tumours are indicated with white dotted ellipses, background tissue (muscle) is indicated with a red arrow. **a** Representative PET images of [^18^F]FDG scan and the last time-frame (40–60 min) after administration of [^11^C]**1** under baseline and blocking conditions, and TACs of tumour under baseline (blue line) and blocking (red line) conditions and of background tissue under baseline (green line) and blocking (yellow line) conditions. **b** Representative PET images of [^18^F]FDG scan and the last time-frame (40–60 min) under baseline conditions, and after blocking (*ERW1041E*) and self-blocking with **2** after administration of [^18^F]**2**, together with TACs of tumour under baseline (blue line) and blocking conditions (red and purple line), and of background tissue under baseline (green line) conditions

Representative images (0–30 min for [^18^F]FDG scans and last time-frame, 40–60 min, for [^11^C]**1** and [^18^F]**2**) and time-activity curves (TAC) of 60 min scans using [^11^C]**1** and [^18^F]**2** under both baseline and blocking conditions (*n* = 4 per group) are depicted in Fig. 4a, b, respectively. The [^18^F]FDG scans revealed rather low uptake in the centres of the tumours, indicating mainly that the rims of the tumours were viable. Under baseline conditions, uptake of [^11^C]**1** in the tumour at baseline conditions showed a peak at 5–10 min after injection (Fig. 4a). Washout, however, was fast and activity concentrations were comparable to those in background tissue (i.e. the muscle, right femur). Furthermore, blocking of TG2 by pretreatment with *ERW1041E* did not result in lower tumour activity levels, but rather resulted in a counterintuitive significant increase in tumour activity, as well as in increased muscle activity concentration. PET scanning using compound [^18^F]**2** showed a time-dependent accumulation in tumour tissue up to 1.7 %ID/g at the 40–60 min time-frame, suggesting irreversible tumour targeting. Background values (the muscle, right femur) reached 0.8 %ID/g at this time-point, which is significantly lower (*p* = 0.0004) and did not display an increase in tissue activity over the scanning period (Fig. 4b). Pretreatment of the animals with the TG2 inhibitor *ERW1041E* resulted in a decrease in activity accumulation in the tumour tissue to 1.4 %ID/g, although this difference was not statistically significant (*p* = 0.06). A drastic and significant decrease in tumour activity concentration, however, was observed when unlabelled **2** was co-administered, to approximately 1.0 %ID/g (*p* = 0.007).

### TG2 expression in MDA-MB-231 tumour tissue—immunohistochemistry and autoradiography on tumour tissue

**Fig. 5:**
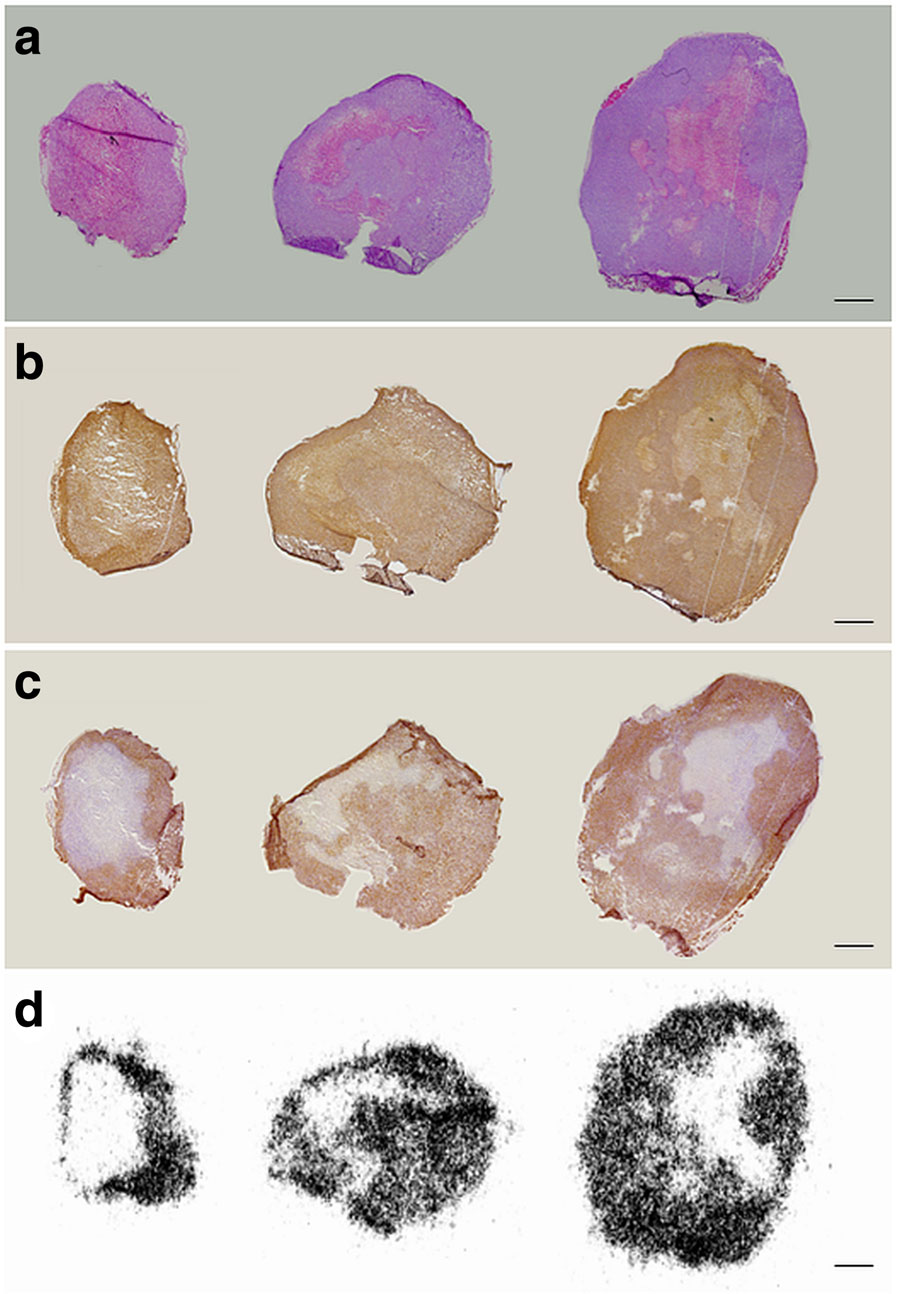
Immunohistochemistry and autoradiography on representative MDA-MB-231 tumour sections (adjacent) obtained from three representative tumours used in this study. **a** Haematoxylin/eosin staining; **b** immunohistochemical detection of TG2; **c** TG2 mediated incorporation of BAP; **d** autoradiography using [^18^F]**2**. Scale bars: 1 mm

Following sacrifice of the animals, tumour sections were evaluated histochemically for TG2 expression and TG2 activity (representative sections of tumour tissues of three separate animals are shown in Fig. 5). In all tumours, haematoxylin/eosin staining (Fig. 5a) demonstrated areas with low nuclei concentrations in the core of the tumour, hinting at central necrosis. Immunohistochemical staining for TG2 expression (Fig. 5b) resulted in a distribution pattern highly resembling the haematoxylin/eosin staining, showing that TG2 is predominantly expressed in the viable part of the tumours. Transglutaminase-mediated BAP incorporation (Fig. 5c) and in vitro autoradiography employing [^18^F]**2** (Fig. 5d), both means of measuring the open (active) conformation of transglutaminase, demonstrated a similar distribution pattern as observed with the anti-TG2 antibody and the haematoxylin/eosin staining, i.e. incorporation of the substrate and inhibitor in the viable areas of tumour tissue.

## Discussion

Transglutaminase mRNA expression in both MDA-MB-231 tumour cells and xenograft tissue essentially was limited to TG2 mRNA and exceeded other transglutaminases by at least a factor ten. The detection of FXIII mRNA in tumour tissue, which is most likely derived from the blood component of the tumour tissue, does not hamper the use of both [^11^C]**1** and [^18^F]**2** TG2 PET tracers, as both compounds show no affinity for activated FXIII [19, 20]. Consequently, these findings imply that the MDA-MB-231 xenograft model is suitable for TG2 PET tracer evaluation.

Previously, metabolic stability of [^11^C]**1** as well as [^18^F]**2** was determined ex vivo in blood plasma of healthy Wistar rats. This study, however, was performed in a xenograft model in SCID mice, and therefore, the metabolic stability of both radiotracers was determined again in SCID mice and results were compared with previously obtained rat data [19, 20]. Metabolism of both [^11^C]**1** and [^18^F]**2** in SCID mice highly resembled metabolism of these compounds in Wister rats. Conversion of [^18^F]**2** to the demethylated analogue **M1**, still a potent TG2 inhibitor, followed the same pattern in both species, although in mice the conversion was less rapid. This difference is likely a result of species-dependent esterase activity [26], and comparable differences between methylester hydrolysis in mice and rats have been reported elsewhere [27]. Importantly, the peptidic backbone and the diazoketone functionality in [^18^F]**2**, responsible for selective and irreversible binding, respectively, appear metabolically stable.

To prevent the development of slow-growing and highly necrotic tumours, an orthotopic mouse model was used, in which tumour cells were inoculated in the 2nd thoracic mammary fat pad [21]. At this position minimal interference with surrounding organs such as liver and heart is expected, thus facilitating definition of ROIs and PET data analysis. The use of the structurally unrelated TG2 inhibitor *ERW1041E* served as a means of determining selectivity of binding of TG2 radiotracers in tumour tissue. *ERW1041E* was administered at 50 mg · kg^−1^, as it has been shown that this dose results in inhibition of TG2 activity to background levels in a mouse model of pulmonary hypertension [9]. In addition, the maximum TG2 inhibitory effect was observed 30 min after *ERW1041E* administration [9].

Compound [^11^C]**1** showed poor tumour uptake and relatively fast washout from the tumour, which was unexpected for an irreversibly binding radiotracer. Therefore, it is believed that the signal is indicative of perfusion rather than TG2 targeting. As both compound **1** and *ERW1041E* inhibit TG2 by irreversibly binding to the active site cysteine residue, a reduction in tumour uptake would be expected upon blocking with *ERW1041E*. The fact that both tumour and muscle activity concentrations increased as a result of *ERW1041E* pretreatment suggests to us a decreased clearance of [^11^C]1 or its derived radioactive metabolites from the blood, although no further experiments were performed to support this claim. Despite the promising results that were previously obtained by in vitro autoradiography, where high specific and selective binding of [^11^C]**1** to MDA-MD-231 tumour sections was observed [19], [^11^C]**1** is not effective as a TG2 PET tracer in vivo. It is unlikely that this apparent ineffectiveness is a result of in vivo metabolism of [^11^C]**1** over the time-course of these experiments, as metabolism is moderate. Potentially, large differences between in vitro TG2 inhibitory potency and inhibition in actual biological systems could explain the inability of this compound to image active TG2 in vivo. Different classes of acryl amide based TG2 inhibitors, although highly potent against isolated TG2, have shown a reduction in inhibitory potency in cell or cell lysate assays [28, 29]. The inhibitory potency of compound **1** in similar cell or cell lysate assays is unknown as such experiments have not been described [30].

In contrast to [^11^C]**1**, [^18^F]**2** displayed higher tumour uptake, which increased over time, indicating irreversible binding of [^18^F]**2** to the tumour tissue. Uptake decreased after pretreating animals with *ERW1041E*, although the difference was not statistically significant (*p* = 0.06). This seemingly partial inhibition of TG2 might be due to the limited TG2 inhibitory potency of *ERW1041E* (K_i_: 11 μM [23]) compared with compound **2**, although previously it has been shown that this compound was able to at least partially inhibit intestinal TG2 in a mouse model [8] and also in a mouse model of pulmonary hypertension to baseline levels [9]. Alternatively, the chosen time-point for pretreatment with *ERW1041E* (set at 30 min prior to tracer administration) might not be optimal for full TG2 inhibition, although at this similar time-point a reduction of TG2 activity by a factor of four in a pulmonary hypertension model was observed [9]. The blocking effect when co-administering unlabelled **2** and [^18^F]**2** (*p* = 0.007), implies that [^18^F]**2** is useful for imaging of local TG2 activity in vivo by indicating specific binding of [^18^F]**2**. However, to establish the selectivity of this uptake, more studies are required.

Analysis of tumour sections showed that TG2 was expressed mainly in the more viable rim of the tumours and less in the necrotic core of the tumours. This decreased tumour viability in the core of the tumours is in close accordance with [^18^F]FDG findings, which generally displayed higher uptake in the outer tumour areas. Because %ID/g was determined by drawing an area over the full tumour volume, areas inside the tumour that contain no tissue transglutaminase potentially underestimate the actual potential of [^18^F]**2** towards TG2 targeting. As PET images using [^18^F]**2** depicted higher activity concentrations in the outer area of the tumour, for future research, it might prove beneficial to perform such an imaging study at an earlier time-point in tumour development, potentially limiting tumour necrosis and thus increasing signal to noise ratios.

Although previously relatively high tissue transglutaminase expression levels in MDA-MB-231 tumour cell lysates were observed [10], and both TG2 expression and activity were further confirmed in xenografted MDA-MB-231 tumour tissue by means of immunohistochemistry and in vitro autoradiography [19, 20], it is unknown to what extent TG2 shows transamidation activity in a biological setting such as the present MDA-MB-231 tumour model. Obviously, only in the open TG2 conformation, active site-directed PET tracers can be successfully applied for TG2 imaging [5, 18]. Therefore, it is expected that primarily extracellular TG2, which is more likely to be in an open conformation due to high extracellular calcium concentrations, will be accessible for such tracers [18]. Previous in vitro studies have demonstrated that TG2 is highly expressed on the plasma membrane of MDA-MB-231 tumour cells [31]. Furthermore, pharmacological inhibition of TG2 on MDA-MB-231 cells using cell-impermeable small molecule inhibitors or antibodies resulted in decreased cell migration [31, 32] and decreased invasiveness [33], which are both hallmarks of cancer [34]. Based on these findings, it is expected that the accumulation of activity in the tumour using [^18^F]**2** is due to targeting of extracellular TG2. Evidence suggests that extracellular TG2 plays a role in tumour progression by means of its transamidation activity, and therefore, the MDA-MB-231 tumour model appears suitable for evaluation of TG2 PET tracers in vivo, which is supported by the imaging results using [^18^F]**2**. Together, the in vivo targeting of TG2 PET tracers is evaluated for the first time and the results suggest that [^18^F]**2** could be used in future research, for example for evaluation of target engagement of other TG2 inhibitors.

## Conclusions

TG2 PET tracers [^11^C]**1** and [^18^F]**2** were evaluated in an MB-MDA-231 breast cancer mouse model. Whereas the TG2 targeting potential of [^11^C]**1** in this model seemed inadequate, [^18^F]**2** showed signs of TG2 targeting, as tumour activity concentrations were steadily increasing over time and could be blocked with TG2 inhibitors.

## Abbreviations

BAP: 5-(Biotinamido)-pentylamine
cDNA: Complementary deoxyribonucleic acid
DAB: 3,3-Diaminobenzidine
DTT: Dithiothreitol
FDG: 2-Fluoro-2-deoxy-d-glucose
FXIII: Blood Coagulation Factor XIII
GAPDH: Glyceraldehyde-3-phosphate-dehydrogenase
GDP: Guanosine diphosphate
GTP: Guanosine triphosphate
HPLC: High-performance liquid chromatography
LC-MS/MS: Liquid chromatography–tandem mass spectrometry
MRM: Multiple reaction monitoring
p.i.: Post injection
PBS: Phosphate buffered saline
PET: Positron emission tomography
qPCR: Quantitative polymerase chain reaction
SPE: Solid phase extraction
TBS: Tris buffered saline
TG: Transglutaminase

## References

[1] Greenberg CS, Birckbichler PJ, Rice RH. Transglutaminases: multifunctional cross-linking enzymes that stabilize tissues. FASEB J. 1991;5:3071–7. 10.1096/fasebj.5.15.1683845 1683845

[2] Gundemir S, Colak G, Tucholski J (1823). Transglutaminase 2: a molecular Swiss army knife. Biochim Biophys Acta Mol Cell Res.

[3] Fesus L, Szondy Z. Transglutaminase 2 in the balance of cell death and survival. FEBS Lett. 2005;579:3297–302. 10.1016/j.febslet.2005.03.063 15943974

[4] Liu S, Cerione RA, Clardy J (2002). Structural basis for the guanine nucleotide-binding activity of tissue transglutaminase and its regulation of transamidation activity. PNAS.

[5] Pinkas DM, Strop P, Brunger AT (2007). Transglutaminase 2 undergoes a large conformational change upon activation. PLoS Biol.

[6] Stamnaes J, Pinkas DM, Fleckenstein B (2010). Redox regulation of transglutaminase 2 activity. J Biol Chem.

[7] Akimov SS, Krylov D, Fleischman LF (2000). Tissue transglutaminase is an integrin-binding adhesion coreceptor for fibronectin. J Cell Biol.

[8] Dafik L, Albertelli M, Stamnaes J (2012). Activation and inhibition of transglutaminase 2 in mice. PLoS One.

[9] DiRaimondo TR, Klöck C, Warburton R (2013). Elevated transglutaminase 2 activity is associated with hypoxia-induced experimental pulmonary hypertension in mice. ACS Chem Biol.

[10] Mehta K, Fok J, Miller FR (2004). Prognostic significance of tissue transglutaminase in drug resistant and metastatic breast cancer. Clin Cancer Res.

[11] Brown KD (2013). Transglutaminase 2 and NF-κB: an odd couple that shapes breast cancer phenotype. Breast Cancer Res Treat.

[12] Klöck C, DiRaimondo TR, Khosla C (2012). Role of transglutaminase 2 in celiac disease pathogenesis. Semin Immunopathol.

[13] Olsen KC, Sapinoro RE, Kottmann RM (2011). Transglutaminase 2 and its role in pulmonary fibrosis. Am J Respir Crit Care Med.

[14] Johnson TS, Griffin M, Thomas GL (1997). The role of transglutaminase in the rat subtotal nephrectomy model of renal fibrosis. J Clin Invest.

[15] Wilhelmus MMM, van Dam A, Drukarch B (2008). Tissue transglutaminase: a novel pharmacological target in preventing toxic protein aggregation in neurodegenerative diseases. Eur J Pharmacol.

[16] De Laurenzi V, Melino G (2001). Gene disruption of tissue transglutaminase. Mol Cell Biol.

[17] Keillor JW, Apperley KYP, Akbar A (2015). Inhibitors of tissue transglutaminase. Trends Pharmacol Sci.

[18] Van der Wildt B, Lammertsma AA, Drukarch B, et al. Strategies towards in vivo imaging of active transglutaminase type 2 using positron emission tomography. Amino Acids. 2016; 10.1007/s00726-016-2288-y. 10.1007/s00726-016-2288-y PMC5332496 27380031

[19] Van der Wildt B, Wilhelmus MMM, Bijkerk J (2016). Development of carbon-11 labeled acryl amides for selective PET imaging of active tissue transglutaminase. Nucl Med Biol.

[20] Van der Wildt B, Wilhelmus MMM, Kooijman EJM (2017). Development of fluorine-18 labelled peptidic PET tracers for imaging active tissue transglutaminase. Nucl Med Biol.

[21] Price JE, Polyzos A, Zhang RD (1990). Tumorigenicity and metastasis of human breast carcinoma cell lines in nude mice. Cancer Res.

[22] Ruijter JM, Ramakers C, Hoogaars WM (2009). Amplification efficiency: linking baseline and bias in the analysis of quantitative PCR data. Nucleic Acids Res.

[23] Watts RE, Siegel M, Khosla C (2006). Structure-activity relationship analysis of the selective inhibition of transglutaminase 2 by dihydroisoxazoles. J Med Chem.

[24] Szanda I, Mackewn J, Patay G (2011). National electrical manufacturers association NU-4 performance evaluation of the PET component of the NanoPET/CT preclinical PET/CT scanner. J Nucl Med.

[25] Nagy K, Tóth M, Major P (2013). Performance evaluation of the small-animal nanoScan PET/MRI system. J Nucl Med.

[26] Bahar FG, Ohura K, Ogihara T (2012). Species difference of esterase expression and hydrolase activity in plasma. J Pharmacol Sci.

[27] Slobbe P, Poot AJ, Haumann R (2016). Two anti-angiogenic TKI-PET tracers, [^11^C]axitinib and [^11^C]nintedanib: radiosynthesis, in vivo metabolism and initial biodistribution studies in rodents. Nucl Med Biol.

[28] Prime ME, Andersen OA, Barker JJ (2012). Discovery and structure−activity relationship of potent and selective covalent inhibitors of transglutaminase 2 for Huntington’s disease. J Med Chem.

[29] Badarau E, Wang Z, Rathbone DL (2015). Development of potent and selective tissue transglutaminase inhibitors: their effect on TG2 function and application in pathological conditions. Chem Biol.

[30] Wityak J, Prime ME, Brookfield FA (2012). SAR development of lysine-based irreversible inhibitors of transglutaminase 2 for Huntington's disease. ACS Med Chem Lett.

[31] Antonyak MA, Li B, Regan AD (2009). Tissue transglutaminase is an essential participant in the epidermal growth factor-stimulated signaling pathway leading to cancer cell migration and invasion. J Biol Chem.

[32] Wang Z, Griffin M (2013). The role of TG2 in regulating S100A4-mediated mammary tumour cell migration. PLoS One.

[33] Mangala LS, Fok JY, Zorrilla-Calancha IR (2007). Tissue transglutaminase expression promotes cell attachment, invasion and survival in breast cancer cells. Oncogene.

[34] Hanahan D, Weinberg RA. The hallmarks of cancer. Cell. 2000;100:57–70. 10.1016/s0092-8674(00)81683-9 10647931

